# LncRNA HCP5 Participates in the Tregs Functions in Allergic Rhinitis and Drives Airway Mucosal Inflammatory Response in the Nasal Epithelial Cells

**DOI:** 10.1007/s10753-022-01620-5

**Published:** 2022-02-05

**Authors:** Chen Yang, Chengfang Shangguan, Changing Cai, Jing Xu, Xiaohua Qian

**Affiliations:** 1 Department of Otolaryngology & Head and Neck Surgery, Ruijin Hospital, Shanghai Jiao Tong University School of Medicine, Huangpu District, 197 Ruijiner Road, Shanghai, 200025 China; 2 Department of Oncology, Ruijin Hospital, Shanghai Jiao Tong University School of Medicine, Huangpu District, Shanghai, 200025 China; 3 School of Biomedical Engineering, Shanghai Jiao Tong University, Xuhui District, 1954 Huashan Road, Shanghai, 200240 China

**Keywords:** allergic rhinitis, long noncoding RNA HCP5, miRNA-16, ATXN2L, regulatory T cells, NECs.

## Abstract

Allergic rhinitis (AR) is an allergic disease characterized as (immunoglobulin, IgE)-mediated type I hypersensitivity disorder. Regulatory T cells (Tregs) play a crucial role in AR. In the present study, we aimed to investigate the mechanism of how Tregs are regulated by long noncoding RNA HCP5 and the regulatory role of HCP5 in IL-13-induced inflammatory response in nasal epithelial cells (NECs) from AR patients. Peripheral blood mononuclear cells (PBMCs) and NECs were obtained from collected blood samples and nasal epithelial tissues. CD4^+^ T cells and Tregs were purified using certain cell isolation kits from PBMCs and Tregs were also differentiated from CD4^+^ T cells using recombinant human IL-2 and TGF-β. The expression levels of HCP5, miR-16, ATXN2L, GM-CSF, eotaxin, and MUC5AC were detected by real-time PCR and western blot. The concentrations of inflammatory cytokines were detected by enzyme-linked immunosorbent assay (ELISA). The interaction among HCP5, miR-16, and ATXN2L were verified by dual-luciferase reporter assay. lncRNA HCP5 expression dramatically downregulated in PBMCs, CD4^+^ T cells, Tregs, and nasal tissues of AR patients, as well as in IL-13-treated NECs. HCP5 promoted Tregs differentiation and proliferation via targeting miR-16/ATXN2L axis. Additionally, HCP5 inhibited IL-13-induced GM-CSF, eotaxin, and MUC5AC production in NECs. HCP5 sponged miR-16 and negatively regulated its expression, and miR-16 targeted ATXN2L and inhibition of miR-16 suppressed IL-13-induced GM-CSF, eotaxin, and MUC5AC expression. HCP5/miR-16/ATXN2L axis mediated Tregs proliferation and functions in AR. Besides, the regulation of IL-13-induced dysfunction of NECs by lncRNA HCP5 depended on miR-16/ATXN2L in the inflammatory response of AR.

**Supplementary Information:**

The online version contains supplementary material available at 10.1007/s10753-022-01620-5.

## INTRODUCTION

Allergic rhinitis (AR), the most common inflammatory disease of the nasal mucosa, is primarily mediated by serum immunoglobulin E (IgE) following contact with allergens and characterized by hypersecretion of mucus [1]. AR has traditionally been considered to originate from a T helper (Th)1/Th2 immune response imbalance, leading to allergic inflammation dominated by the Th2 immune response within the nasal mucosa [2]. Following further study, the pathogenesis of AR has been extended from the Th1/Th2 model to a Th1/Th2/Th17 and T regulatory cell (Treg) model [3]. However, as AR is a multi-factor disease induced by gene-environment interactions, its exact pathogenesis has not been elucidated.

The long noncoding RNAs (lncRNAs) are defined as a group of single-stranded RNA with lengths longer than 200 bp that are not translated into protein [4]. lncRNAs reside in the nucleus or cytoplasm and interact with nucleic acids or proteins, and have been shown to regulate dosage compensation, genomic imprinting, pluripotency, cell differentiation and development, immune response, etc. lncRNAs bring about such copious functions by employing diverse mechanisms such as translational inhibition, mRNA degradation, RNA decoys, regulation of protein activity, and regulating the availability of miRNAs by sponging mechanism [5]. Long non-coding RNAs (lncRNAs) have been proven to play important roles in a variety of human immune diseases. For example, LincRNA GATA3-AS1 might serve as a specific indicator of Th2 response and Th2-associated diseases and might be involved in Th2 cell differentiation [6]. The lncRNAs have also been implicated in AR. A lncRNA microarray analysis showed that a total of 2259 lncRNAs including 1033 up-regulated and 1226 down-regulated were significantly differentially expressed in the nasal mucosa samples from 4 AR patients as compared to those from 4 non-allergic subjects [7]. However, their pathological effects on the development of allergic rhinitis (AR) have not been clearly understood.

In the present study, we previously have utilized GEO datasets (including GSE159415, GSE100386, GSE50223, and GSE140454) to conduct bioinformatics analysis of the gene expression profiling data, and demonstrated that lncRNA HCP5 was lowly expressed in AR patients compared with healthy volunteers. Histocompatibility leukocyte antigen complex P5 (HCP5), initially reported in 1993, is an important lncRNA located between the MICA and MICB genes in MHC I region [4]. HCP5 is primarily found expressed in immune system cells and had a potential role in autoimmunity [8]. Treg cells play important roles in the maintenance of immune homeostasis [9]. Therefore, in the present study, we aimed to investigate the effect of HCP5 in Tregs differentiation and function during AR development. Besides, IL-13 is a typical Th2 cytokine that has been shown to be the central mediator of physiologic changes induced by allergic inflammation, and has been implicated in mucus hypersecretion and inflammatory mediator release by airway epithelial cells [10]. IL-13-treated NECs, which have been shown to play an essential role in the innate immune response to AR, is a commonly used cell model for AR analysis [11]. Herein, we also explored the function of HCP5 on IL-13-induced inflammatory cytokine and mucus production in NECs from AR patients. Thus, our findings would provide the basis for potential clinical applications of HCP5 in AR prevention and treatment.

## MATERIALS AND METHODS

### Patients and Samples

Total of 30 AR patients and 30 healthy volunteers were recruited in the Ruijin Hospital, Shanghai Jiao Tong University School of Medicine from January 2019 to December 2020. Nasal mucosal tissue samples were obtained surgically from the inferior turbinate from 30 patients with perennial AR and 30 healthy volunteers. All samples were stored at −20 ℃ until use. All patients in the AR group had a positive skin-prick test (SPT) to dust mites, animal dander, cockroaches, and/or molds and a positive screening of specific IgE. Each AR patient was diagnosed based on his/her medical history, nasal endoscopic examination, an allergen skin-prick test, and a serum specific IgE assay. None of the patients had received topical or systemic corticosteroid therapy for 4 weeks prior to study recruitment. The subjects with chronic rhinosinusitis, nasal polyps, and infectious rhinitis; a history of smoking; and/or other immune system disorders, such as rheumatoid arthritis, systemic lupus erythematosus, and scleroderma, were excluded in this study. This study was approved by the Ethic Committee of Shanghai Jiao Tong University School of Medicine, and written consent was derived from all the participants.

### RNA Extraction and qRT-PCR Validation

Total RNA was isolated from PBMCs, CD4^+^ T cells, Tregs, NECs, and nasal mucosal tissues using TRIzol Reagent following the manufacturer’s protocol. Total RNA was reversely transcribed into cDNA using PrimeScript RT Master Mix (TaKaRa, Dalian, China) following the manufacturer’s instructions. qRT-PCR was performed by using SYBR Premix Ex Taq II (TaKaRa) on the 7900 HT Sequence Detection System (ABI, USA). Glyceraldehyde 3-phosphate dehydrogenase (GAPDH) and U6 were used as the endogenous controls of lncRNA or mRNAs and microRNA. The comparative threshold cycle (2^−ΔΔCt^) method was used to detect relative expression levels. Sequences of primers used for qRT-PCR were as followed in the Table S1.

### Isolated and Purification of PBMCs, Tregs, and CD4^+^ T Cells

Peripheral blood mononuclear cells (PBMCs) were isolated from the whole blood samples of healthy volunteers and AR patients. Then, Tregs were purified using the Tregs-specific cell isolation kits (Miltenyi Biotec) according to the manufacturer’s instruction. Tregs were then stimulated using the anti-CD3/CD28 beads (Invitrogen) at the ratio of 1:1 cell-to-bead coculture for 36 h, and then added rhIL-2 and TGF-β for another 4-day incubation in RPMI-1640 medium suppling with 10% fetal calf serum, sodium pyruvate, L-glutamine, 100 IU/mL penicillin, and 100 μg/mL streptomycin, at 37 °C with 5% CO_2_. The whole blood CD4 micro-beads (Miltenyi Biotec) were used to isolate CD4^+^ T cells following the manufacturer’s protocol. After isolation, CD4^+^ T purity was > 95%.

### Tregs Differentiation

According to the description in the previous study [12], the isolated CD4^+^ T cells (7 × 10^5^ cells/mL) were also stimulated using the anti-CD3/CD28 beads (Invitrogen) at the ratio of 1:1 cell-to-bead coculture for 36 h, and rhIL-2 and TGF-β were then added for additional 4-day incubation in RPMI-1640 medium suppling with 10% fetal calf serum, sodium pyruvate, L-glutamine, 100 IU/mL penicillin, and 100 μg/mL streptomycin, at 37 °C with 5% CO_2_.

### NEC Culture and Treatment

Primary NECs were isolated and cultured as described previously [13]. Inferior turbinate NECs were collected by nasal scraping under local anesthesia. The NECs were then cultured in BEGM medium (Lonza, Walkersville, MD, USA) under submerged conditions. When reaching 80–90% confluence, the NECs were passaged. Subsequently, the NECs were stimulated with or without 50 ng/mL IL-13 for 24 h. Cell supernatant and pellets were then harvested for analysis.

### Cell Transfections

To knockdown of HCP5, a special small interference RNA (siRNA) containing a 19-bp interfering sequence against HCP5 transcript was transfected into cells using Lipofectamine 3000 (Invitrogen), and pcDNA3.1-HCP5 or control plasmids from RiboBio were transfected into cells to establish the HCP5-overexpression cells. miR-16 mimic, inhibitor, miRNA control, human recombinant ATXN2L (OE-ATXN2L), and negative control (1 μM) were purchased from RiboBio Co., Ltd. (Guangzhou, China). Sequences of these specific targets were listed in the Table S2.

### Flow Cytometry

The percentage of Tregs among CD4^+^ T cells was detected using flow cytometry. A total of 1 × 10^6^ cells (mixed Tregs and CD4^+^ T) were first stained with surface markers. The intracellular staining was then performed on the cells using the Tregs Staining Kit (eBioscience) following the manufacturer’s instructions. Flow cytometry was conducted using the BD Fortessa (BD Bioscience).

### ELISA

The concentrations of IL-2, IL-10, IFN-γ, TGF-β1, IL-4, IL-5, and IL-17 in different cells were detected by the commercial ELISA kits from Shanghai Enzyme-linked Biotechnology Co., Ltd. (Shanghai, China) following the manufacturer’s protocol.

### Dual-Luciferase Reporter Assay

To evaluate the interaction between HCP5 and miR-16, or miR-16 and ATXN2L, Tregs or NECs were transfected with psiCHECK2-based constructs containing HCP5 Wild-Type (HCP5-WT) and HCP5-Mutant (HCP5-MUT). The mutated 3’-UTR of HCP5 or ATXN2L containing the putative miR-16-binding site were predicted by the website, amplified by PCR, and generated using the QuikChangeIIsSite-Directed Mutagenesis Kit (Agilent). Cells were seeded in 24-well plates and then co-transfected with 50-nM miR-16 mimics or miR-NC and 0.2-μg luciferase reporter plasmid using Lipofectamine 3000 (Thermo Fisher). After 48 h, firefly and *Renilla* luciferase activity was examined by the Dual-Luciferase Reporter Assay System (Promega) and data were normalized against values of co-transfected *Renilla* luciferase.

### Cell Counting Kit-8

After transfections, Tregs (2 × 10^3^ cells/well) in 96-well plates containing 200 µL of culture medium was cultivated in 37 °C incubator with 5% CO_2_. Twenty microliters of CCK8 solution (5 mg/mL) was added to each well of the plate to measure changes in cell viability. This plate was then incubated at 37 °C for an additional 2 h. Absorbance at 450 nm was evaluated under a microreader. The experiment was performed with three replicates.

### Western Blot Assay

The protein from cells and tissue samples were lysed using a RIPA lysis buffer (Beyotime Biotechnology, China) with protease inhibitor (Roche, Basel, Switzerland) and phosphatase inhibitor cocktails (Bimake, China). Followed by ultrasound for 30 s and centrifuged at 12,000 rpm for 20 min, the concentration of protein was quantified using a BCA Protein Assay Kit (Thermo Scientific). The total protein (35 μg) was separated by 10% sodium dodecyl sulfate–polyacrylamide gel electrophoresis and transferred onto a PVDF membrane (Millipore). The membrane was blocked in 5% nonfat milk in TBST for 1 h at room temperature and then incubated primary antibodies overnight at 4 °C. The membranes were washed three times and then incubated with corresponding horseradish peroxidase-labeled secondary antibodies for 1 h at 37 °C. The signal was developed with an ECL Blotting Substrates (Bio-Rad). The densitometry was quantified and analyzed using ImageJ software.

### Statistical Analysis

All data were presented as mean ± standard deviation (SD). Data were analyzed by 2-way analysis of variance or Student’s *t* test analysis and one-way ANOVA analysis. All data were determined in triplicate and are representative of at least two separate experiments. The correlation between lncRNA HCP5, miR-16, and ATXN2L were analyzed using the Pearson correlation analysis. The statistical difference was considered as significant when *p* value is < 0.05.

## RESULTS

### LncRNA HCP5 Was Lowly Expressed in AR Patients

**Fig. 1:**
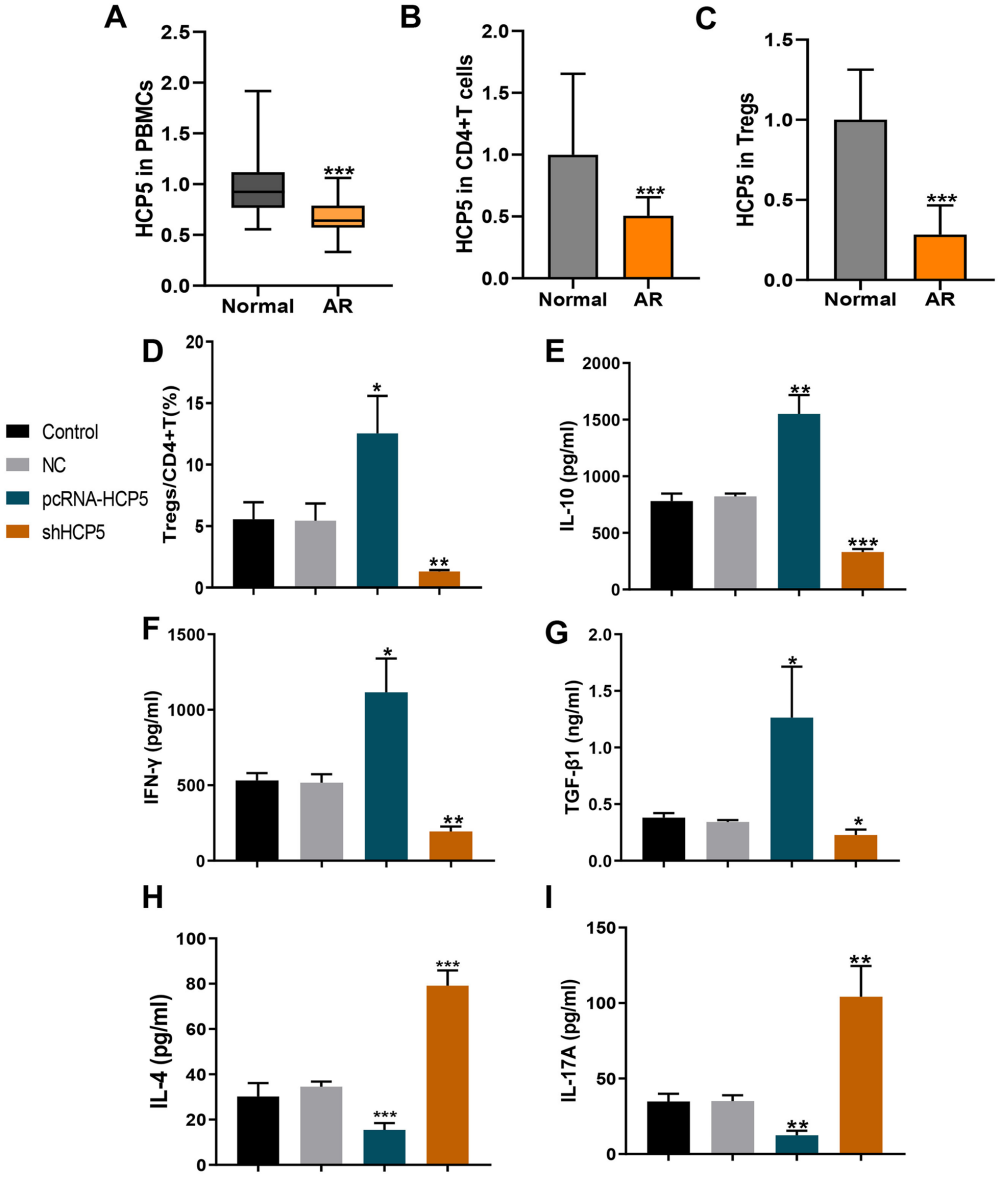
Overexpression of lncRNA HCP5 in peripheral blood mononuclear cells (PBMCs), CD4^+^ T cells, and Tregs cells from AR patients. (**A**) HCP5 expression in PBMCs from 30 AR patients and 30 healthy volunteers were measured by qRT-PCR. (**B** and **C**) qRT-PCR analysis of the relative mRNA expressions of HCP5 in CD4^+^ T cells and Tregs cells from AR patients or healthy volunteers. ***p < 0.001, compared to normal. (**D**) The addition of HCP5 overexpression upregulated the proportion of Treg cells in CD4^+^ subsets compared to the PHA-stimulated PBMC group (control group), while the knockdown of HCP5 was in contrast. (**E**–**I**) The addition of HCP5 overexpression led to the downregulation of IL-10, IFN-γ, and TGF-β1 levels and the upregulation of IL-4 and IL-17A levels, while the knockdown of HCP5 was in contrast. The data represent the mean ± SD. Experiments were repeated in triplicate: *p < 0.05, **p < 0.01, and ***p < 0.001, compared to the PHA-stimulated PBMC vehicle group healthy controls (Control).

To explore the effect of HCP5 in AR, we first detected the mRNA expression level of HCP5 in the PBMCs, CD4^+^ T cells, and Tregs isolated from the peripheral blood of 30 pairs of AR patients (AR) and healthy volunteers (Normal), respectively. As shown in Fig. 1A, HCP5 expression level was much lower in the PBMCs of AR patients than that in normal volunteers. Moreover, in the CD4^+^ T cells, the HCP5 level significantly decreased in AR group when compared with normal group (Fig. 1B). Furthermore, the HCP5 level in Tregs of the AR group dramatically downregulated comparing to normal (Fig. 1C). These results demonstrated the association of lncRNA HCP5 and AR and the potential role of lncRNA HCP5 in Tregs function.

### LncRNA HCP5 Promoted Tregs Differentiation

We further detected the role of lncRNA HCP5 in Tregs differentiation. Tregs proportion significantly increased after HCP5 overexpression treatment, while dramatically decreased after HCP5 knockdown (Fig. 1D) compared with the negative control (NC), suggesting that HCP5 could promote Tregs differentiation. The effect of HCP5 on Tregs-regulated inflammatory cytokines was further detected. The concentrations of IL-10, IFN-γ, and TGF-β1 were significantly increased after transfection with pcDNA3.1-HCP5 while decreased after HCP5 siRNA administration compared with NC group (Fig. 1E–G). In contrast, some other related cytokines such as IL-4 and IL-17 obviously decreased upon the HCP5 treatment, while increased after HCP5 silencing (Fig. 1 H and I). The results from ELISA suggested that lncRNA HCP5 impacted the immune imbalance *in vitro*.

### miR-16 Was Highly Expressed in AR Patients and Inhibited Tregs Differentiation

**Fig. 2:**
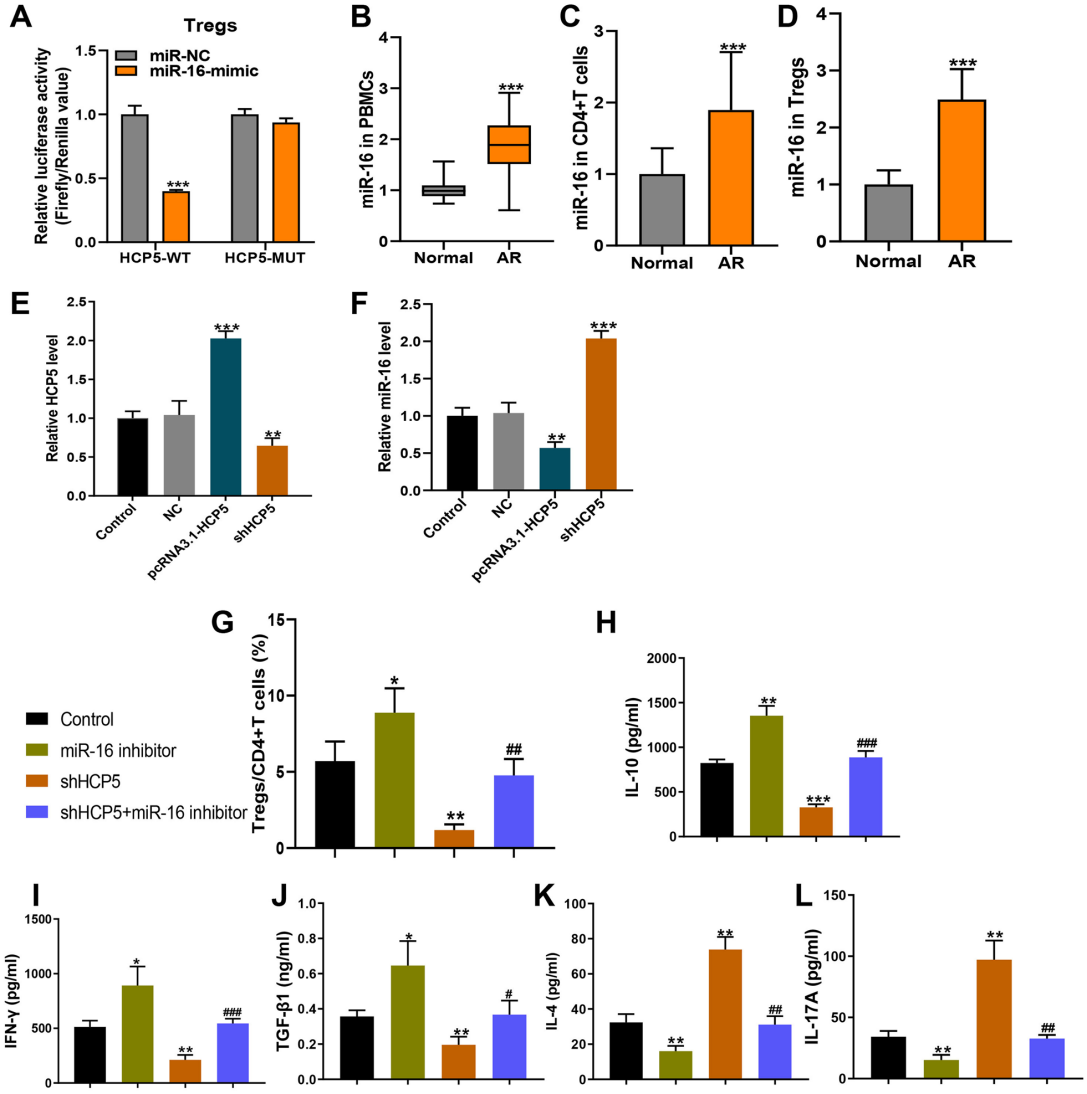
LncRNA HCP5 negatively regulated miR-16 as an RNA sponge. (**A**) The luciferase activities in Treg cells co-transfected with miR-16 or miR-NC mimics and luciferase reporters containing HCP5-wt or HCP5-mt. Data are presented as the relative ratio of hRluc luciferase activity to hluc + luciferase activity. (**B**–**D**) qRT-PCR analysis of the relative mRNA levels of miR-16 in PBMC cells, CD4^+^ T cells, and Treg cells from AR patients or healthy volunteers. (**E** and **F**) The expressions of HCP5 and miR-16 in the sorted Tregs transfected with pcDNA3.1-HCP5 or shHCP5 were detected by RT-qPCR. (**G**) The addition of miR-16 inhibitor upregulated the proportion of Treg cells in CD4^+^ subsets, while the addition of shHCP5 weakened the effect of miR-16 inhibitor. (**H**–**L**) The addition of miR-16 inhibitor led to the upregulation of IFN-γ, IL-10, and TGF-β1 levels and the downregulation of IL-4 and IL-17A levels, while the addition of shHCP5 weakened the effect of miR-16 inhibitor. The data represent the mean ± SD. Experiments were repeated in triplicate: *p < 0.05, **p < 0.01, and ***p < 0.001 vs. Control. #*p* < 0.05, ##*p* < 0.01, and ###*p* < 0.001 vs. shHCP5.

First, the target of lncRNA HCP5 was further predicted online and we found that miR-16 was predicted to bind to HCP5. To validate whether miR-16 was a functional target of lncRNA HCP5, we utilized the dual-luciferase reporter system. As shown in Fig. 2A, the HCP5-WT or MUT was cloned into the luciferase reporter vector and co-transfected with miR-16 mimics or miR-NC into Tregs. Luciferase assay results showed that miR-16 mimics significantly decreased the luciferase activity of the reporter gene with WT but not HCP5-MUT construct, suggesting that miR-16 may be the functional target of lncRNA HCP5. Therefore, we next detected the expression levels of miR-16 in 30 AR patients. As shown in Fig. 2B–D, miR-16 expression level significantly increased both in the PBMCs, CD4^+^ T cells, and Tregs of AR patients when compared with control healthy volunteers.

To test whether HCP5 regulated miR-16, we overexpressed HCP5 in Tregs and then monitored the miR-16 level. As shown in Fig. 2E, transfecting siRNA against HCP5 significantly decreased the level of HCP5. Meanwhile, knockdown of HCP5 resulted in significantly increased level of miR-16 (Fig. 2F). In contrast, overexpression of HCP5 by transfecting pcDNA3.1-HCP5 resulted in significantly increased HCP5 level while significantly decreased the miR-16 level (Fig. 2E, F). These results demonstrated that HCP5 negatively regulated miR-16 expression in Tregs.

### HCP5/miR-16 Regulated Tregs Differentiation and Proliferation

To further explore the effect of miR-16 in AR and Tregs differentiation, we transfected Tregs with miR-16 inhibitor. As shown in Fig. 2G, miR-16 inhibitor dramatically enhanced Tregs percentage in CD4^+^ T cells, which is opposite to the effect of HCP5 knockdown. However, additional miR-16 inhibitor administration based on HCP5 knockdown significantly promoted Tregs differentiation *in vitro*. Additionally, miR-16 inhibitor dramatically enhanced the concentrations of IFN-γ, IL-10, and TGF-β1, while inhibited the concentrations of IL-4 and IL-17A, which is opposite to the role of HCP5 knockdown (Fig. 2H–L). Expectedly, additional miR-16 inhibitor administration significantly attenuated the HCP5 effect in Tregs-associated cytokine production. These findings indicated that HCP5 influenced the immune imbalance and promoted Tregs differentiation by sponging miR-16 *in vitro*.

**Fig. 3:**
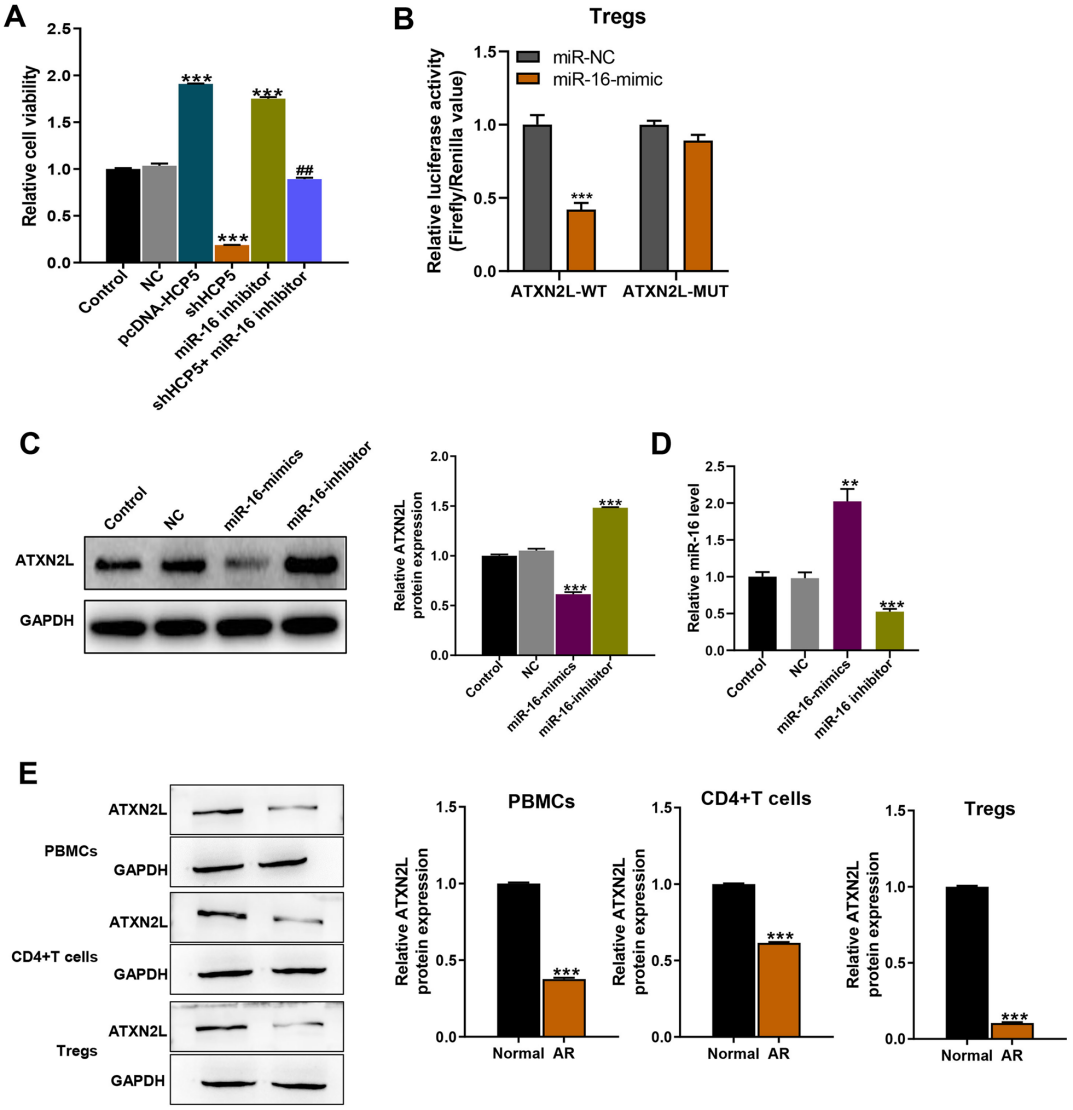
miR-16/ATXN2L regulated function in Tregs. (**A**) The proliferation of Tregs after treated by PHA (Control), miR-NC, pcDNA3.1-HCP5, shHCP5, miR-16 inhibitor, and shHCP5 + miR-16 inhibitor. (**B**) The luciferase activities in Treg cells co-transfected with miR-16 or miR-NC mimics and luciferase reporters containing ATXN2L-wt or ATXN2L-mt. Data are presented as the relative ratio of hRluc luciferase activity to hluc + luciferase activity. (**C** and **D**) The expressions of ATXN2L in the sorted Tregs transfected with miR-16 inhibitor or miR-16 mimics were detected by western blot and RT-qPCR. (**E**) Western blot analysis of the relative expression of ATXN2L in PBMCs, CD4^+^ T cells, and Tregs cells from AR patients or healthy controls. The data represent the mean ± SD. Experiments were repeated in triplicate: **p < 0.01 and ***p < 0.001, compared to Control group.

To further investigate the effect of HCP5/miR-16 on the proliferation of Tregs, we isolated Tregs from human PBMCs and treated with control, NC, pcDNA3.1-HCP5, siHCP5, miR-16 inhibitor, and siHCP5 + miR-16 inhibitor. As presented in Fig. 3A, HCP5 overexpression and miR-16 inhibitor significantly enhanced Tregs proliferation, while HCP5 knockdown reduced Tregs proliferation. Interestingly, based on HCP5 silence, additional miR-16 inhibitor administration remarkably promoted Tregs proliferation.

### ATXN2L Expression Decreased in AR Patients

We continued to explore the underlying functional mechanism of miR-16 and used starBase to predict the target of miR-16. We also performed dual-luciferase reporter system to further confirm the functional interaction between miR-16 and ATXN2L. Luciferase assay results showed that expression of miR-16 mimics significantly decreased the luciferase activity of the reporter gene with WT but not MUT of ATXN2L 3′UTR construct in Tregs (Fig. 3B). Besides, ATXN2L protein expression in miR-16 mimics transfected Tregs significantly decreased while increased in miR-16 inhibitor group (Fig. 3C), indicating that ATXN2L might be the downstream target underlying miR-16. Recognizing the results above, we next detected the expression levels of ATXN2L in AR patients. As shown in Fig. 3E, ATXN2L protein expression level significantly decreased in PBMCs, CD4^+^ T cells, and Tregs from AR patients when compared with control healthy volunteers. All the results demonstrated that ATXN2L expression levels decreased in AR patients.

### ATXN2L Promoted Tregs Differentiation and Proliferation

**Fig. 4:**
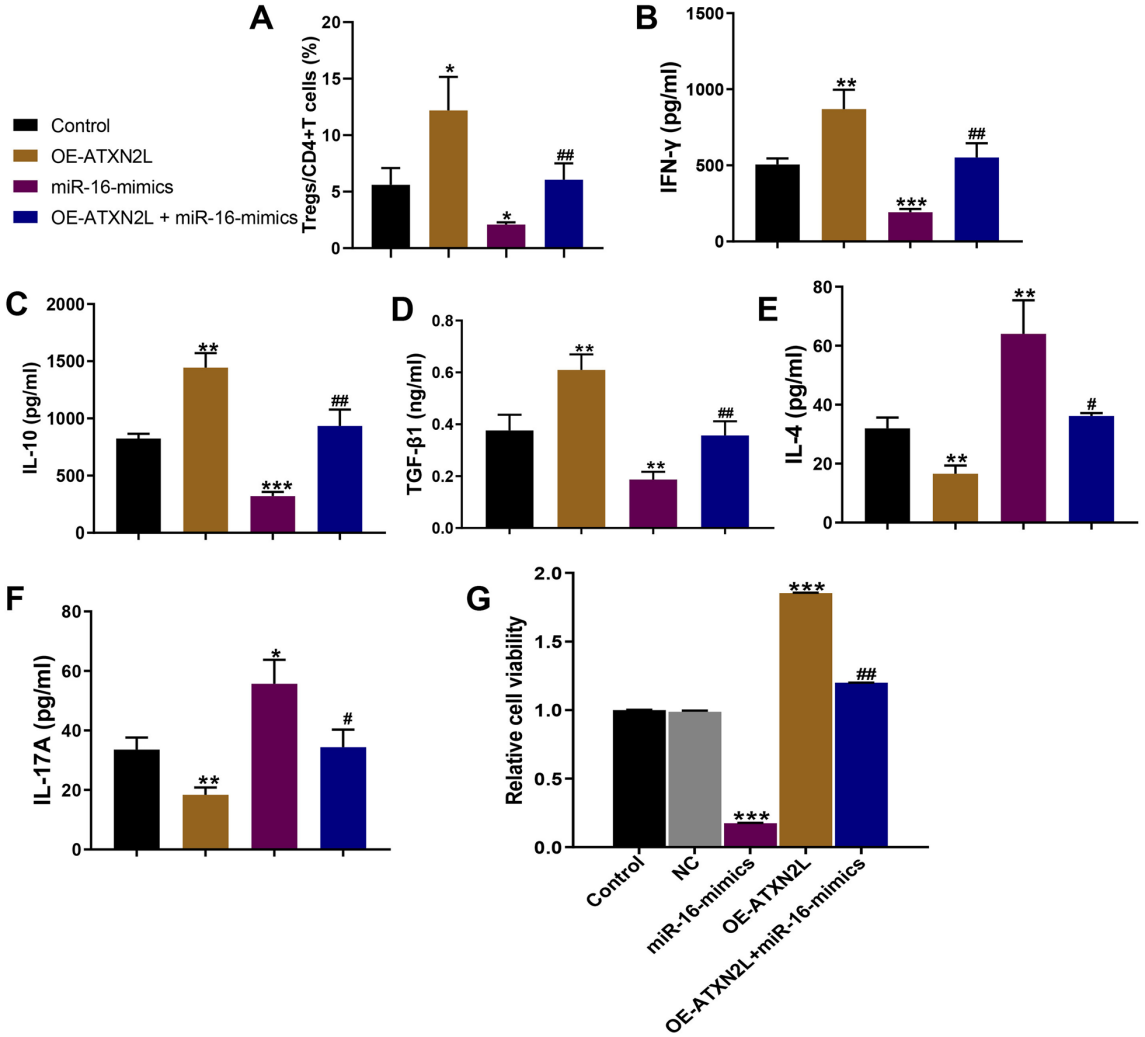
miR-16/ATXN2L impacted the immune imbalance and promoted the differentiation of Treg cells *in vitro*. (**A**) The addition of ATXN2L upregulated the proportion of Treg cells in CD4^+^ subsets, while the addition of miR-16 weakened the effect of ATXN2L. (**B**–**F**) The addition of ATXN2L led to the upregulation of IFN-γ, IL-10, and TGF-β1 levels and the downregulation of IL-4 and IL-17A levels, while the addition of miR-16 weakened the effect of ATXN2L. The data represent the mean ± SD. Experiments were repeated in triplicate: **p < 0.01, compared to the PHA-stimulated PBMC vehicle group (Control group); #*p* < 0.05 and ##*p* < 0.01 vs. miR-16 mimics. (**G**) The proliferation of Tregs after treated by PHA (Control), miR-NC, miR-16 mimics, OE-ATXN2L, and OE-ATXN2L + miR-16 mimics.

We transfected Tregs with miR-16 inhibitor or mimics and found that the miR-16 expression was significantly decreased or increased in Tregs (Fig. 3D). To further explore the effect of ATXN2L in Tregs differentiation, we first added OE-ATXN2L (1 μM) in CD4^+^ T cells *in vitro* for 1 h at 37 °C. Flow cytometry showed that ATXN2L overexpression dramatically enhanced Tregs percentage in CD4^+^ T cells, which is opposite to the effect of miR-16. However, additional miR-16 mimics administration based on ATXN2L significantly suppressed Tregs differentiation (Fig. 4A). Besides, ATXN2L dramatically enhanced the concentrations of IFN-γ, IL-10, and TGF-β1, while it inhibited the concentrations of IL-4 and IL-17A, which is opposite to the role of miR-16 mimics (Fig. 4B–F). In addition, additional miR-16 mimics administration significantly attenuated the miR-16 effect on Tregs-associated cytokine production (Fig. 4B–F). Importantly, miR-16 mimics significantly suppressed Tregs proliferation while ATXN2L obviously enhanced Tregs proliferation. However, based on the ATXN2L treatment, additional miR-16 administration obviously inhibited proliferating Tregs (Fig. 4G). These data demonstrated that ATXN2L impacted the immune imbalance and promoted the differentiation of Treg cells *in vitro*, and MiR-16/ATXN2L seems to be involved in the regulation of Tregs proliferation and function.

### Expression of lncRNA HCP5/miR-16/ATXN2L in AR Patients and IL-13-Treated NECs

**Fig. 5:**
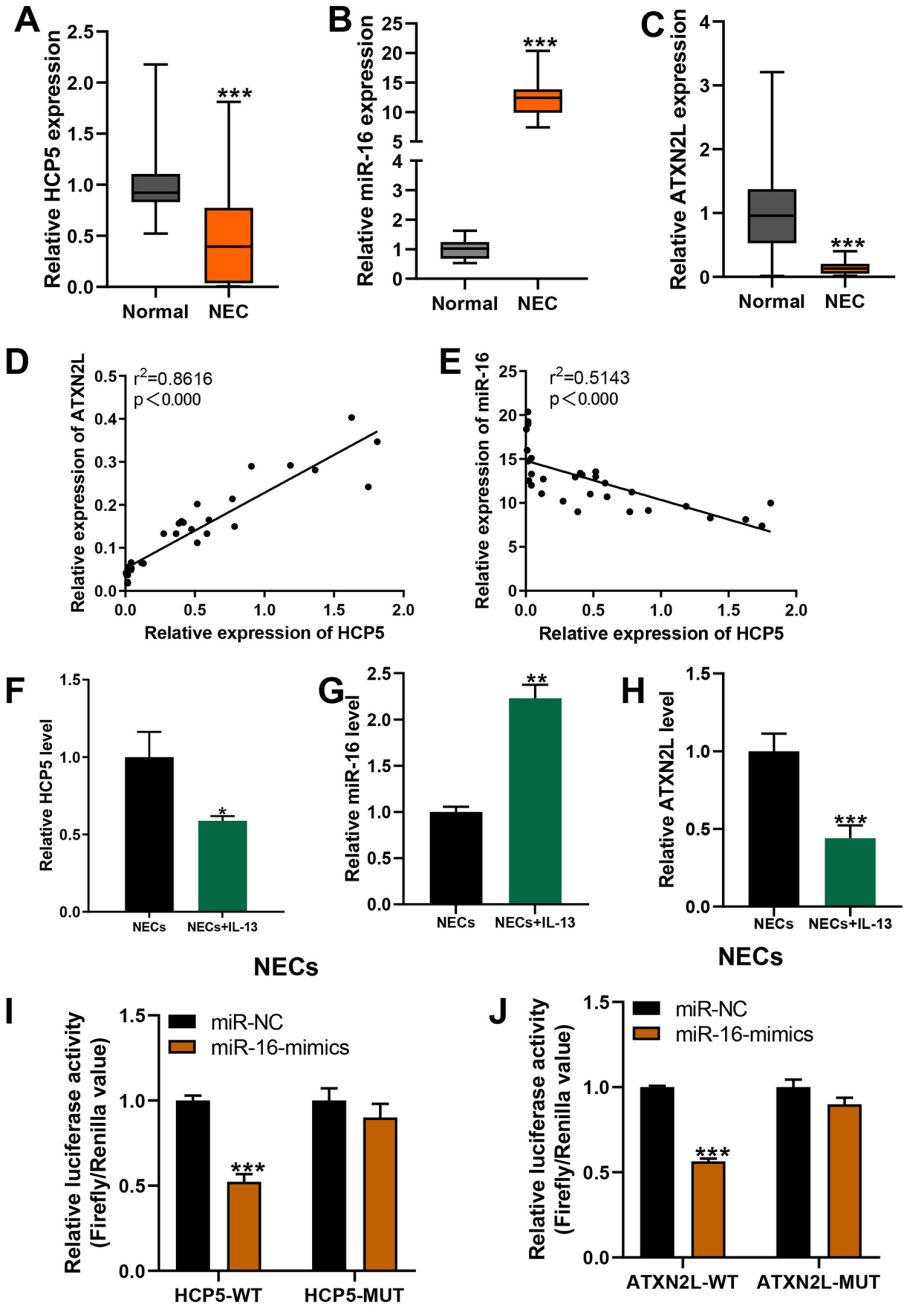
lncRNAHCP5/miR-16/ATXN2L level in the nasal epithelial cells (NECs). (**A**–**C**) The expression levels of HCP5, miR-16, and ATXN2L in NECs from AR patients or healthy volunteers were measured by qRT-PCR. (**D** and **E**) Correlation analysis between HCP5 and ATXN2L, or HCP5 and miR-16 in NECs from 30 AR patients. (**F**–**H**) The expression levels of HCP5, miR-16, and ATXN2L in NECs after IL-13 stimulation (50 ng/mL) for 24 h were measured by qRT-PCR. *p < 0.05, **p < 0.01, and ***p < 0.001. (**I** and **J**) The luciferase activities in Treg cells co-transfected with miR-16 or miR-NC mimics and luciferase reporters containing ATXN2L-wt or ATXN2L-mt, and HCP5-wt or HCP5-mt. Data are presented as the relative ratio of hRluc luciferase activity to hluc + luciferase activity.

To further investigate the potential role of lncRNA HCP5/miR-16/ATXN2L in AR, we evaluated their expression levels in the nasal mucosal tissues from AR patients. As shown in Fig. 5 A and C, HCP5 and ATXN2L were significantly downregulated in the nasal mucosal tissue samples from AR patients when compared to samples from healthy people. However, miR-16 was obviously increased in the nasal tissues of AR patients when compared with normal (Fig. 5B). We performed Pearson correlation analysis to evaluate the expression relationship between lncRNA HCP5 and miR-16, as well as lncRNA HCP5 and ATXN2L. We found that HCP5 expression level was significantly negatively correlated with miR-16 expression level while HCP5 was significantly positively correlated with ATXN2L (Fig. 5 D and E).

Based on the evidence that IL-13 was so much related to the pathogenesis of AR, the model of IL-13 induction was widely accepted in the study of AR [14], so in this study, we treated NECs with IL-13. After NEC isolation, we also detected significantly decreased HCP5 and ATXN2L while increased miR-16 in IL-13-treated NECs when compared to untreated cells (Fig. 5F–H).

### LncRNA HCP5 Negatively Regulated miR-16, Which Directly Targeting ATXN2L in NECs

The bind sites of miR-16 on HCP5 and ATXN2L have been predicted through the online software starBase and miRcode before. The wild-type or mutant of HCP5 or ATXN2L were cloned into the luciferase reporter vector and co-transfected with miR-16 mimics or miRNA-NC into the NECs. Luciferase assay results showed that expression of miR-16 mimics significantly decreased the luciferase activity of the reporter gene with wild type but not mutated HCP5 or ATXN2L construct (Fig. 5 I and J). These findings demonstrated that HCP5 could regulate NEC function by targeting miR-16/ATXN2L.

### LncRNA HCP5 Suppressed Inflammatory Response in NECs

**Fig. 6:**
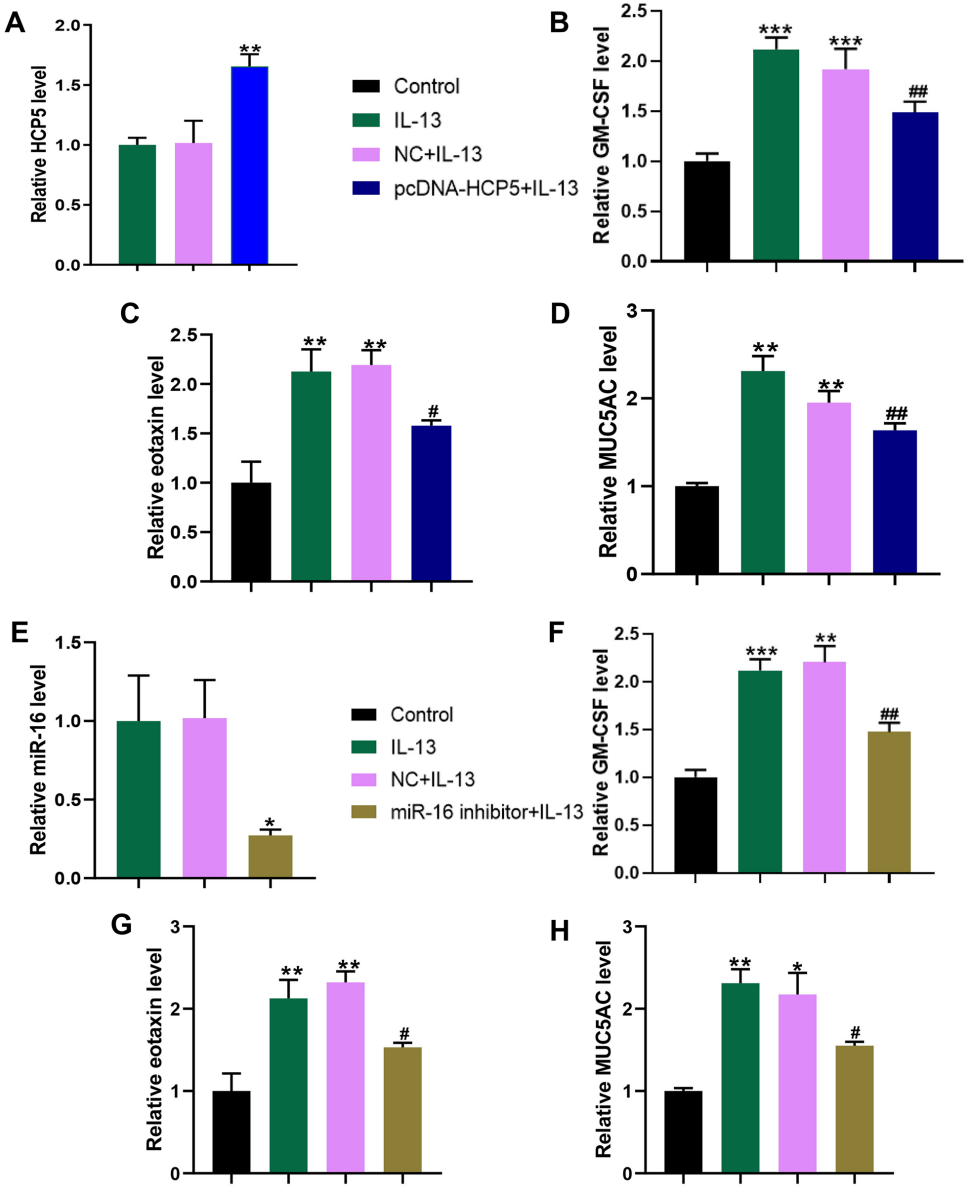
Effects of HCP5/miR-16 on IL-13-induced GM-CSF, eotaxin, and MUC5AC expression in NECs. (**A**) The knockdown efficiency of pcDNA3.1-HCP5 in NECs was measured by qRT-PCR. (**B**–**D**) Effects of HCP5 overexpression on IL-13-induced inflammatory cytokine and mucin mRNA expression in cultured NECs. The mRNA expression levels of GM-CSF, eotaxin, and MUC5AC were measured by qRT-PCR. (**E**) The knockdown efficiency of miR-16 inhibitor in NECs was measured by qRT-PCR. (F–H) Effects of miR-16 inhibitor on IL-13-induced inflammatory cytokine and mucin mRNA expression in cultured NECs. The mRNA expression levels of GM-CSF, eotaxin, and MUC5AC were measured by qRT-PCR. The data represent the mean ± SD. Experiments were repeated in triplicate: *p < 0.05, **p < 0.01, and ***p < 0.001 vs. Control; #*p* < 0.05 and ##*p* < 0.01 vs. IL-13.

GM-CSF and eotaxin are pro-inflammatory cytokines in airway epithelial cells during allergic airway inflammation. MUC5AC is a glycoprotein belonging to the superfamily of mucins and mucus hypersecretion (particularly MUC5AC expression) is a common feature of allergic airway disorders [14]. To overexpress HCP5, we transfected NECs with the plasmid pcDNA3.1-HCP5, which encoded the HCP5 expression. We detected significantly increased HCP5 levels in NECs when compared to NECs transfected with NC vector (Fig. 6A). The mRNA levels of GM-CSF (Fig. 6B) and eotaxin (Fig. 6C) were significantly increased in NECs treated with 50 ng/mL IL-13 for 24 h. In contrast, GM-CSF and eotaxin mRNA levels were significantly decreased in HCP5 overexpressing NECs when compared to NC transfected cells (Fig. 6B, C). Similarly, MUC5AC mRNA level was significantly increased in NECs treated with IL-13 and overexpression of HCP5 significantly decreased MUC5AC mRNA level (Fig. 6D). Collectively, our data indicated that HCP5 inhibited IL-13-induced GM-CSF, eotaxin, and MUC5AC expression in NECs.

### Inhibition of miR-16 Prevented IL-13-Induced GM-CSF, Eotaxin, and MUC5AC Expression in NECs

We next evaluated the potential roles of miR-16 in AR using the IL-13-treated NEC model. We transfected NECs with miR-16 inhibitor and found that the miR-16 expression was significantly decreased in IL-induced NECs (Fig. 6E). The inhibition of miR-16 significantly prevented IL-13-induced mRNA levels of GM-CSF (Fig. 6F), eotaxin (Fig. 6G), and MUC5AC (Fig. 6H). In conclusion, our results indicated that miR-16 regulated the expressions of GM-CSF, eotaxin, and MUC5AC in NECs, suggesting the potential roles of miR-16 in AR.

### LncRNA HCP5 Mediated IL-13-Induced Dysfunction of NECs via Regulating miR-16/ATXN2L Expression

**Fig. 7:**
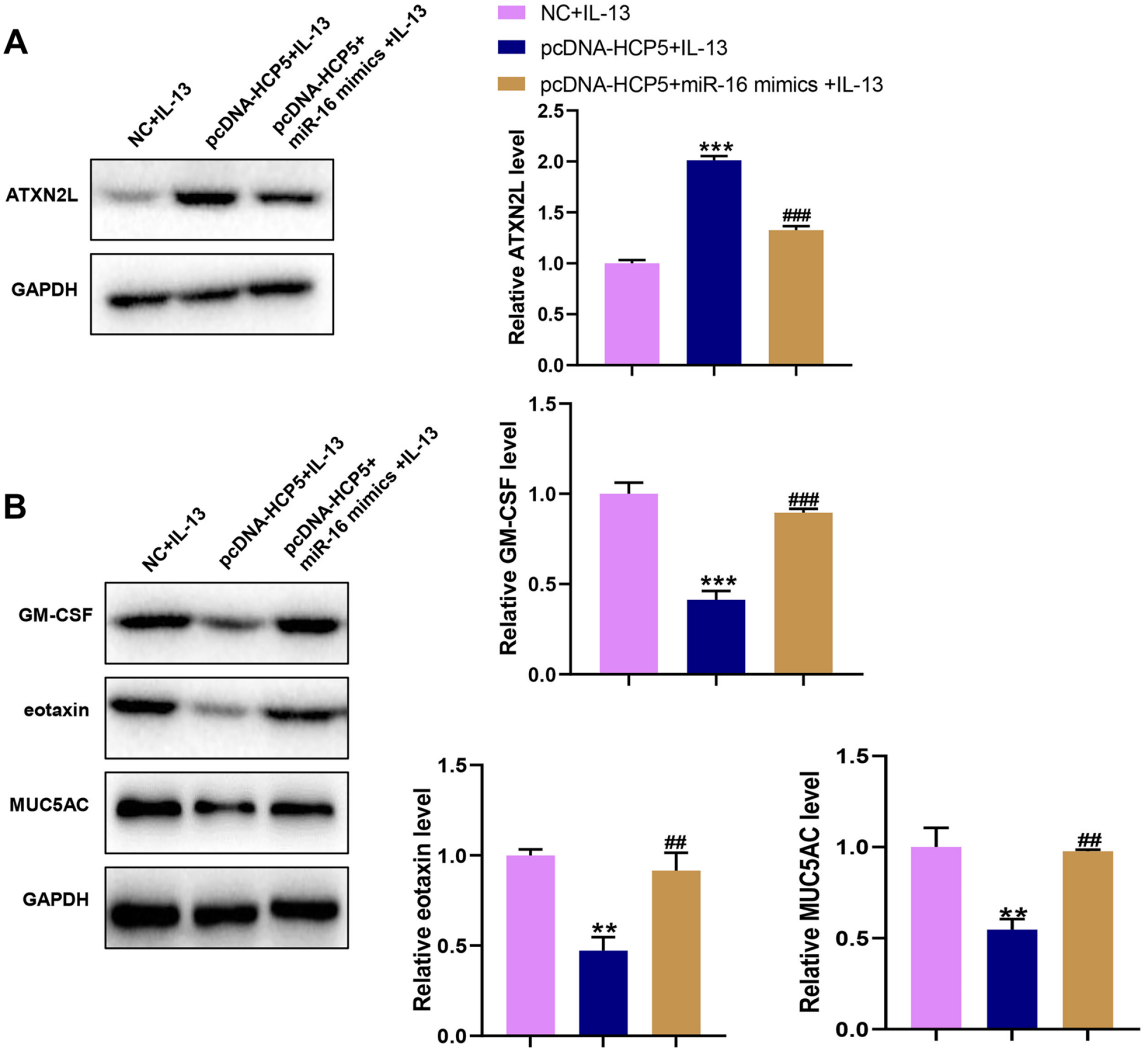
Effects of HCP5/miR-16/ATXN2L on IL-13-induced GM-CSF, eotaxin, and MUC5AC protein expression in NECs. (**A**) The protein levels of ATXN2L in NECs co-transfected with pcDNA3.1-HCP5 and pcDNA3.1-HCP5 + miR-16 mimics. (**B**) miR-16 reverted the decreased IL-13-induced inflammatory cytokine and mucin mRNA expression in cultured NECs due to HCP5 overexpression. The protein expression levels of GM-CSF, eotaxin, and MUC5AC were measured by western blot. The data represent the mean ± SD. Experiments were repeated in triplicate: **p < 0.01 and ***p < 0.001 vs. NC + IL-13; ##*p* < 0.01 and ###*p* < 0.001 vs. pcDNA-HCP5 + IL-13.

Due to HCP5 negatively regulated miR-16, we further explored whether the inhibitory effect of HCP5 on IL-13-induced dysfunction and positive regulation on ATXN2L depended on miR-16. We overexpressed both HCP5 and miR-16 in NECs. We found that miR-16 significantly inhibited HCP5-induced ATXN2L overexpression at protein level (Fig. 7A). In addition, miR-16 rescued the protein expressions of GM-CSF, eotaxin, and MUC5AC (Fig. 7B), which were inhibited by HCP5 in IL-13-treated NECs. Therefore, our results demonstrated that HCP5 mediated IL-13-induced dysfunction of NECs via regulating miR-16/ATXN2L expression.

## DISCUSSION

The prevalence of allergic rhinitis (AR) has increased worldwide in recent decades [15]. In the present study, we found that the lowly expressed lncRNA HCP5 in PBMCs, CD4^+^ T cells, and Tregs from AR patients regulates the differentiation and function of Tregs via regulating miR-16/ATXN2L. Moreover, the concentrations of Tregs-related cytokines altered after HCP5 overexpression or knockdown treatment, as well as after the alteration of miR-16 and ATXN2L in Tregs. Furthermore, our study also indicated that HCP5 and ATXN2L were strongly downregulated in nasal mucosal tissues from AR patient while miR-16 was notably upregulated. Additionally, it had shown that HCP5 targeted miR-16 and negatively regulated miR-16 expression while positively regulated ATXN2L expression in Tregs and primary NECs from AR patients. Interestingly, HCP5 suppressed the expressions of GM-CSF, eotaxin, and MUC5AC in IL-13-treated NECs while miR-16 promoted these gene expressions. Besides, miR-16 targeted ATXN2L and inhibited ATXN2L expression. More importantly, overexpression of miR-16 abolished the inhibitory effects of HCP5 on IL-13-induced expression of GM-CSF, eotaxin, and MUC5AC. Therefore, our findings demonstrated the regulatory role of lncRNA HCP5/miR-16/ATXN2L axis in inflammatory response of IL-13-induced NECs in AR.

LncRNA dysregulation underlies certain human diseases caused by chromosome deletion and translocation [16]. Numbers of specific lncRNAs have been identified in Th1 and Th2 cells [6, 17, 18] and in the nasal mucosa of mice with AR [15], for instance, Linc00632 in IL-13-induced inflammatory cytokine and mucus production in NECs from AR patients [13]. However, to the best of our knowledge, no published studies on the mechanism associated with lncRNAHCP5 function in AR are currently available. LncRNA HCP5 is mainly expressed in the immune system and often considered to be associated with herpes zoster and other serious skin reactions [8]. Recent studies have reported differential expression of HCP5 in multiple cancers [4, 19, 20]. Herein, HCP5 was obviously downregulated in AR patients, and promoted Tregs percentage in CD4^+^ T cells and the concentrations of Tregs-associated cytokines such as IFN-γ, IL-10, and TGF-β1, while enhanced the expression of Th2 cytokines, IL-4 and IL-17A. As expected, HCP5 knockdown showed the opposite effect of Tregs differentiation and proliferation, as well as the cytokine levels. We further demonstrated that HCP5 inhibited IL-13-induced GM-CSF, eotaxin, and MUC5AC production, indicating a protective role of HCP5 in AR.

The target-mimetic, sponge/decoy function of lncRNA on miRNAs has been recently uncovered [21]. Though there were many reports about HCP5 act as miRNA sponge [19, 20, 22], it was the first time to explore the precise role of HCP5 as the miRNA sponge in AR. Our findings identified that HCP5 targeted miR-16 and negatively its expression. Panganiban et al. identified 30 miRNAs that were differentially expressed among healthy, AR, and asthmatic subjects, and found that miR-16 was upregulated in AR and asthmatic patients versus healthy volunteers [23]. Circulatory miR-16 levels are also one of the most predictive signs of allergy and asthma status [24], which was consistent with our findings that miR-16 was upregulated in AR patients when compared with normal people. Recently, the functions of miR-16 that have been reported are mainly in cancer field. For example, highly expressed miR-16 represses the viability and proliferation, and promotes their apoptosis in cervical cancer cells [25]. Serum miR-16 level was significantly decreased in hepatocellular carcinoma patients and exists anticancer activity [26]. Moreover, miR-16 plays an important role in shifting macrophage polarization from M2 to M1 status, and functionally activating CD4( +) T cells [27]. In our study, miR-16 inhibitor administration dramatically enhanced Tregs percentage in CD4^+^ T cells and the concentrations of Tregs-associated cytokines. Moreover, miR-16 inhibitor attenuated the inhibiting role of HCP5 deletion in Tregs proliferation and function. What is more, we demonstrated that the regulation of IL-13-induced GM-CSF, eotaxin, and MUC5AC production by HCP5 depended on miR-16. This is the first description of miR-16 function in AR. LncRNA HCP5 may sponge miR-16 to regulate the immune inflammation in the nasal mucosa to prevent AR.

The main role of miRNAs is to regulate mRNA expression levels of target genes through directing the degradation or modulating transcriptional repression of target mRNA [28]. The biological function of ATXN2L, a gene known to be directly related to allergic diseases, was found to be associated with decreased allergy risk in a genome-wide association study (GWAS) [29]. Besides, the cellular ATXN2 concentration was important for the assembly of stress granules and P-bodies, which are main compartments for regulating and controlling mRNA degradation, stability, and translation [30]. In addition, ATXN2L promotes cell invasiveness and oxaliplatin resistance and can be upregulated by EGF via PI3K/Akt signaling in gastric cancer [31]. However, no study reported the effect of ATXN2L in AR before. We further identified that miR-16 targeted ATXN2L and the current study demonstrated that ATXN2L expression levels decreased in PBMCs, CD4^+^ T cells, and Tregs of AR patients. ATXN2L administration dramatically enhanced Tregs percentage in CD4^+^ T cells and the concentrations of Tregs-associated cytokines. Moreover, ATXN2L attenuated the inhibiting role of miR-16 in Tregs proliferation and function. Besides, HCP5 negatively regulated IL-13-induced GM-CSF, eotaxin, and MUC5AC production via miR-16/ATXN2L axis in human NECs. Therefore, targeting ATXN2L and its downstream factors could be a promising strategy to treat AR.

It is important to note the limitations of this study. All data in our study were from an *in vitro* study. The mouse model of AR should be used to further mimic the AR progress and detect the biological role of HCP5/miR-16/ATXN2L in AR development.

## CONCLUSION

Collectively, we explored the biological role of HCP5/miR-16/ATXN2L in Tregs differentiation and function in AR. We provided evidence that lncRNA HCP5 expression dramatically downregulated in PBMCs, CD4^+^ T cells, Tregs, and IL-13-treated NECs of AR patients. HCP5/miR-16/ATXN2L are associated with regulatory T-cell differentiation and function, and regulated IL-13-induced inflammatory cytokine and mucus production in allergic rhinitis.

## Supplementary Information

Below is the link to the electronic supplementary material.

Supplementary file1 (DOCX 17 KB)

Supplementary file2 (DOCX 16 KB)

## Data Availability

The datasets used and/or analyzed during the present study are available from the corresponding author on reasonable request.

## References

[1] Seidman, M.D., R.K. Gurgel, S.Y. Lin, S.R. Schwartz, F.M. Baroody, and J.R. Bonner, et al. 2015. Clinical practice guideline: allergic rhinitis executive summary. *Otolaryngology--head and neck surgery : official journal of American Academy of Otolaryngology-Head and Neck Surgery*. 152(2):197–206. 10.1177/0194599814562166. 10.1177/0194599814562166 25645524

[2] Eifan AO, Durham SR (2016). Pathogenesis of rhinitis. Clinical and experimental allergy : Journal of the British Society for Allergy and Clinical Immunology..

[3] Milner JD (2011). IL-17 producing cells in host defense and atopy. Current opinion in immunology..

[4] Zou, Y., and B. Chen. 2021. Long non-coding RNA HCP5 in cancer. *Clinica chimica acta; international journal of clinical chemistry*. 512:33–9. 10.1016/j.cca.2020.11.015. 10.1016/j.cca.2020.11.015 33245911

[5] Akhade VS, Pal D, Kanduri C (2017). Long noncoding RNA: Genome organization and mechanism of action. Advances in experimental medicine and biology..

[6] Zhang H, Nestor CE, Zhao S, Lentini A, Bohle B, Benson M (2013). Profiling of human CD4+ T-cell subsets identifies the TH2-specific noncoding RNA GATA3-AS1. The Journal of allergy and clinical immunology..

[7] Ma Z, Teng Y, Liu X, Li J, Mo J, Sha M (2017). Identification and functional profiling of differentially expressed long non-coding RNAs in nasal mucosa with allergic rhinitis. The Tohoku Journal of Experimental Medicine..

[8] Liu Y, Helms C, Liao W, Zaba LC, Duan S, Gardner J (2008). A genome-wide association study of psoriasis and psoriatic arthritis identifies new disease loci. PLoS genetics..

[9] Zhou L, Chong MM, Littman DR (2009). Plasticity of CD4+ T cell lineage differentiation. Immunity.

[10] Ingram, J.L., and M. 2012. Kraft. IL-13 in asthma and allergic disease: asthma phenotypes and targeted therapies. *The Journal of allergy and clinical immunology*. 130(4):829–42; quiz 43–4. 10.1016/j.jaci.2012.06.034. 10.1016/j.jaci.2012.06.034 22951057

[11] Haenuki Y, Matsushita K, Futatsugi-Yumikura S, Ishii KJ, Kawagoe T, Imoto Y (2012). A critical role of IL-33 in experimental allergic rhinitis. The Journal of allergy and clinical immunology..

[12] Wang L, Yang X, Li W, Song X, Kang S (2019). MiR-202-5p/MATN2 are associated with regulatory T-cells differentiation and function in allergic rhinitis. Human cell..

[13] Yue L, Yin X, Hao F, Dong J, Ren X, Xu O (2020). Long noncoding RNA Linc00632 inhibits interleukin-13-induced inflammatory cytokine and mucus production in nasal epithelial cells. Journal of innate immunity..

[14] Teng Y, Zhang R, Liu C, Zhou L, Wang H, Zhuang W (2015). miR-143 inhibits interleukin-13-induced inflammatory cytokine and mucus production in nasal epithelial cells from allergic rhinitis patients by targeting IL13Rα1. Biochemical and Biophysical Research Communications.

[15] Wang XD, Zheng M, Lou HF, Wang CS, Zhang Y, Bo MY (2016). An increased prevalence of self-reported allergic rhinitis in major Chinese cities from 2005 to 2011. Allergy.

[16] Batista PJ, Chang HY (2013). Long noncoding RNAs: Cellular address codes in development and disease. Cell.

[17] Spurlock CF, Tossberg JT, Guo Y, Collier SP, Crooke PS, Aune TM (2015). Expression and functions of long noncoding RNAs during human T helper cell differentiation. Nature Communications.

[18] Collier, S.P., P.L. Collins, C.L. Williams, M.R. Boothby, and T.M. Aune. 2012. Cutting edge: influence of Tmevpg1, a long intergenic noncoding RNA, on the expression of Ifng by Th1 cells. *Journal of immunology* (Baltimore, Md : 1950). 189(5):2084–8. 10.4049/jimmunol.1200774. 10.4049/jimmunol.1200774 PMC3424368 22851706

[19] Chen Y, Zhang X, An Y, Liu B, Lu M (2020). LncRNA HCP5 promotes cell proliferation and inhibits apoptosis via miR-27a-3p/IGF-1 axis in human granulosa-like tumor cell line KGN. Molecular and cellular endocrinology..

[20] Yuan B, Guan Q, Yan T, Zhang X, Xu W, Li J (2020). LncRNA HCP5 regulates pancreatic cancer progression by miR-140-5p/CDK8 axis. Cancer biotherapy & radiopharmaceuticals..

[21] Paraskevopoulou MD, Hatzigeorgiou AG (2016). Analyzing MiRNA-LncRNA interactions. Methods in molecular biology (Clifton, NJ)..

[22] Zhu K, Wang L, Zhang X, Sun H, Chen T, Sun C (2020). LncRNA HCP5 promotes neuroblastoma proliferation by regulating miR-186-5p/MAP3K2 signal axis. Journal of pediatric surgery..

[23] Panganiban RP, Wang Y, Howrylak J, Chinchilli VM, Craig TJ, August A (2016). Circulating microRNAs as biomarkers in patients with allergic rhinitis and asthma. The Journal of allergy and clinical immunology..

[24] Specjalski K, Jassem E (2019). MicroRNAs: Potential biomarkers and targets of therapy in allergic diseases?. Archivum immunolgiae et therapiae experimentalis.

[25] Ding Z, Liu SJ, Liu XW, Ma Q, Qiao Z (2020). MiR-16 inhibits proliferation of cervical cancer cells by regulating KRAS. European review for medical and pharmacological sciences..

[26] Bashir AO, El-Mesery ME, Anwer R, Eissa LA (2020). Thymoquinone potentiates miR-16 and miR-375 expressions in hepatocellular carcinoma. Life sciences..

[27] Jia X, Li X, Shen Y, Miao J, Liu H, Li G (2016). MiR-16 regulates mouse peritoneal macrophage polarization and affects T-cell activation. Journal of cellular and molecular medicine..

[28] Bartel DP (2004). MicroRNAs: Genomics, biogenesis, mechanism, and function. Cell.

[29] Ferreira MAR, Vonk JM, Baurecht H, Marenholz I, Tian C, Hoffman JD (2019). Eleven loci with new reproducible genetic associations with allergic disease risk. The Journal of allergy and clinical immunology..

[30] Nonhoff U, Ralser M, Welzel F, Piccini I, Balzereit D, Yaspo ML (2007). Ataxin-2 interacts with the DEAD/H-box RNA helicase DDX6 and interferes with P-bodies and stress granules. Molecular biology of the cell..

[31] Lin L, Li X, Pan C, Lin W, Shao R, Liu Y (2019). ATXN2L upregulated by epidermal growth factor promotes gastric cancer cell invasiveness and oxaliplatin resistance. Cell Death & Disease.

