# Supplementary material for: LncRNA HCP5 Participates in the Tregs Functions in Allergic Rhinitis and Drives Airway Mucosal Inflammatory Response in the Nasal Epithelial Cells
Source: Inflammation. 2022 Feb 5;45(3):1281–97. doi: 10.1007/s10753-022-01620-5 (PMC9095562; doi:10.1007/s10753-022-01620-5)
Supplement: Supplementary file 1 — Supplementary file1 (DOCX 17 KB) [file 10753_2022_1620_MOESM1_ESM.docx]

| **Gene name** | **Forward sequence (5’-3’)** | **Reverse sequence (5’-3’)** |
| --- | --- | --- |
| HCP5 | GACTCTCCTACTGGTGCTTGGT | CACTGCCTGGTGAGCCTGTT |
| ATXN2L | GGAGCCGAAGGCATCTTGG | TCTGGAATTGTTGTAGACGCC |
| GM-CSF | TCCTGAACCTGAGTAGAGACAC | TGCTGCTTGTAGTGGCTGG |
| eotaxin | CCCCTTCAGCGACTAGAGAG | TCTTGGGGTCGGCACAGAT |
| MUC5AC | CAGCACAACCCCTGTTTCAAA | GCGCACAGAGGATGACAGT |
| GAPDH | GAACGGGAAGCTCACTGG | GCCTGCTTCACCACCTTCT |
| U6 | CTCGCTTCGGCAGCACA | AACGCTTCACGAATTTGCGT |
| miR-16 | **Sequence**: 5’-GTCAGCAGTGCCTTAGCAG-3’ | |

**Supplementary Table 1. Sequences of primers used for qRT-PCR.**

| **Gene name** | **Forward sequence (5’-3’)** | **Reverse sequence (5’-3’)** |
| --- | --- | --- |
| HCP5 | GACTCTCCTACTGGTGCTTGGT | CACTGCCTGGTGAGCCTGTT |
| ATXN2L | GGAGCCGAAGGCATCTTGG | TCTGGAATTGTTGTAGACGCC |
| GM-CSF | TCCTGAACCTGAGTAGAGACAC | TGCTGCTTGTAGTGGCTGG |
| eotaxin | CCCCTTCAGCGACTAGAGAG | TCTTGGGGTCGGCACAGAT |
| MUC5AC | CAGCACAACCCCTGTTTCAAA | GCGCACAGAGGATGACAGT |
| GAPDH | GAACGGGAAGCTCACTGG | GCCTGCTTCACCACCTTCT |
| U6 | CTCGCTTCGGCAGCACA | AACGCTTCACGAATTTGCGT |
