# Supplementary material for: LncRNA HCP5 Participates in the Tregs Functions in Allergic Rhinitis and Drives Airway Mucosal Inflammatory Response in the Nasal Epithelial Cells
Source: Inflammation. 2022 Feb 5;45(3):1281–97. doi: 10.1007/s10753-022-01620-5 (PMC9095562; doi:10.1007/s10753-022-01620-5)
Supplement: Supplementary file 2 — Supplementary file2 (DOCX 16 KB) [file 10753_2022_1620_MOESM2_ESM.docx]

**Supplementary Table 2. Sequences of specific targets.**

| **Name** | **Sequence (5’-3’)** |
| --- | --- |
| siHCP5 (Forward) | GCAAUAGACUGAGAUGCAATT |
| siHCP5 (Reverse) | UUGCAUCUCAGUCUAUUGCTT |
| negative control (Forward) | UUCUCCGAACGUGUCACGUTT |
| negative control (Reverse) | ACGUGACACGUUCGGAGAATT |
| HCP5-WT | CCCGAACCCUCCUCCUGCUGU |
| HCP5-MUT | CCCGAACCCUCCUGGACGACU |
| miR-16 inhibitor | CACCAAUAUUUACGUGCUGCUA |
| inhibitor-NC | CAGUACUUUUGUGUAGUACAA |
| miR-16 mimics | UAGCAGCACGUAAAUAUUGGCG |
| miR-NC (Forward) | UUCUCCGAACGUGUCACGUTT |
| miR-NC (Reverse) | ACGUGACACGUUCGGAGAATT |
